# Environmental and occupational respiratory diseases – 1057. Correlation between air pollution and respiratory health of school children in Delhi

**DOI:** 10.1186/1939-4551-6-S1-P55

**Published:** 2013-04-23

**Authors:** Jincy Mathew, Radha Goyal, KK Taneja, Naveen Arora

**Affiliations:** 1 Allergy and Immunology Section, Institute of Genomics and Integrative Biology, Delhi, India; 2 Air Pollution, National Environmental Engineering Research Institute, Delhi Zonal Lab, India; 3 Lipid Biochemistry, Institute of Genomics and Integrative Biology, Delhi, India

## Background

Urban air pollution (indoor and outdoor) poses a major health hazard for city dwellers. Exposure to ambient air pollution may result in respiratory health disorders. The aim of this study was to correlate air pollutant levels to the respiratory health of school children in Delhi.

## Methods

The questionnaire was designed based on the internationally valid questionnaires for respiratory illness. The respiratory health survey was approved by the Directorate of Education – Delhi and schools were selected based on land usage pattern i.e. commercial (Chandni Chowk), industrial (Mayapuri) and residential (Sarojini Nagar) areas. Approximately 1800 students (600 / zone) of age 10 – 14 years participated in the survey which included spirometry tests also. Indoor and outdoor levels of SO_2_, NO_2_ and PM were also measured in the school premises.

## Results

The questionnaire data showed that the students having respiratory disorder symptoms was maximum in Chandni Chowk (66%) followed by Mayapuri (59%) and Sarojini Nagar (46%). Spirometry test results demonstrated that a significant population of subjects in Chandni Chowk (19%) had mild to severe pulmonary obstruction. However the percentage of subjects with such conditions was comparatively less in Maypuri (17%) and Sarojini Nagar (14%) area. Indoor and outdoor PM_10_ concentration at schools located in Chandni Chowk, a commercial zone, was observed to be 815±354.45 µg/m^3^ & 337±85 µg/m^3^ respectively, which is ten times above the permissible limits. The PM_10_ concentration was lower in Mayapuri (694.6±322.9 µg/m^3^ & 274±78 µg/m^3^), an industrial zone, and was least in Sarojini Nagar (534.3±94.22µg/m^3^ & 197±48 µg/m^3^) which is a residential zone. However, levels of SO_2_ and NO_2_were under the permissible limits in all three areas.

## Conclusions

There is a correlation between pollutant levels and symptoms of respiratory illness in children. Commercial zones like Chandni Chowk with high traffic movement and human activities contribute more PM which affects the pulmonary health of children.

